# COVID-19 induces neuroinflammation and loss of hippocampal neurogenesis

**DOI:** 10.21203/rs.3.rs-1031824/v1

**Published:** 2021-10-29

**Authors:** Robyn Klein, Allison Soung, Cheick Sissoko, Anna Nordvig, Peter Canoll, Madeline Mariani, Xiaoping Jiang, Traci Bricker, James Goldman, Gorazd Rosoklija, Victoria Arango, Mark Underwood, J. John Mann, Adrianus Boon, Andrew Dowrk, Maura Boldrini

**Affiliations:** Washington University School of Medicine; Washington University School of Medicine; Columbia University; Weill Cornell Medicine; Columbia University Medical Center; Columbia University; Washington University School of Medicine; Washington University School of Medicine; Columbia University; Columbia University; Columbia University; Columbia University; Washington University; Columbia University; Columbia University

**Keywords:** COVID-19, neuroinflammation, central nervous system symptoms

## Abstract

Infection with the Severe Acute Respiratory Syndrome Coronavirus 2 (SARS-CoV-2) is associated with onset of neurological and psychiatric symptoms during and after the acute phase of illness^1–4^. Acute SARS-CoV-2 disease (COVID-19) presents with deficits of memory, attention, movement coordination, and mood. The mechanisms of these central nervous system symptoms remain largely unknown.

In an established hamster model of intranasal infection with SARS-CoV-2^5^, and patients deceased from COVID-19, we report a lack of viral neuroinvasion despite aberrant BBB permeability, microglial activation, and brain expression of interleukin (IL)-1β and IL-6, especially within the hippocampus and the inferior olivary nucleus of the medulla, when compared with non-COVID control hamsters and humans who died from other infections, cardiovascular disease, uremia or trauma. In the hippocampus dentate gyrus of both COVID-19 hamsters and humans, fewer cells expressed doublecortin, a marker of neuroblasts and immature neurons.

Despite absence of viral neurotropism, we find SARS-CoV-2-induced inflammation, and hypoxia in humans, affect brain regions essential for fine motor function, learning, memory, and emotional responses, and result in loss of adult hippocampal neurogenesis. Neuroinflammation could affect cognition and behaviour via disruption of brain vasculature integrity, neurotransmission, and neurogenesis, acute effects that may persist in COVID-19 survivors with long-COVID symptoms.

During the COVID-19 pandemic, mounting evidence indicated SARS-CoV-2 infection leads to central nervous system (CNS) dysfunction^6,7^. During acute COVID-19, patients without prior neuropsychiatric history have trouble concentrating, poor sleep, fatigue, hallucinations, delusions, and behavioural changes^8^. Moreover, an alarmingly high fraction of patients, perhaps as high as 33%, continue to suffer neuropsychiatric symptoms post-hospital discharge, including a dysexecutive syndrome consisting of inattention, disorientation, and poor movement coordination^2,3,9–13^. Postmortem human neuropathological findings in COVID-19 include hypoxic damage, microglial activation, astrogliosis, leukocytic infiltration and microhemorrhages^14–16^, suggesting that, at least in some cases, the CNS undergoes neuropathological sequelae associated with hypoxia and neuroinflammation. This is supported by neuroimaging studies in post-acute COVID-19 patients, showing disruption of fractional anisotropy and diffusivity, suggesting micro-structural and functional alterations of the hippocampus^17^, a brain region critical for memory formation, and part of a conserved subcortical network involved in anxiety and stress responses.

Thus far, the neurobiological bases of COVID-19 neuropsychiatric symptoms remain largely unknown, and no studies have assessed the human hippocampus postmortem. Here, we investigated CNS changes associated with SARS-CoV-2 infection in Golden Syrian hamsters (*Mesocricetus auratus*), and in humans deceased from severe COVID-19, compared with non-COVID control hamsters and age and sex-matched humans (Table 1) who died of other infections (39%), cardiovascular disease (46%), uremia (8%) and trauma (8%).

## Hamsters intranasally infected with SARS-CoV-2 and patients deceased from acute COVID-19 do not exhibit viral neuroinvasion

**Figure 1:**
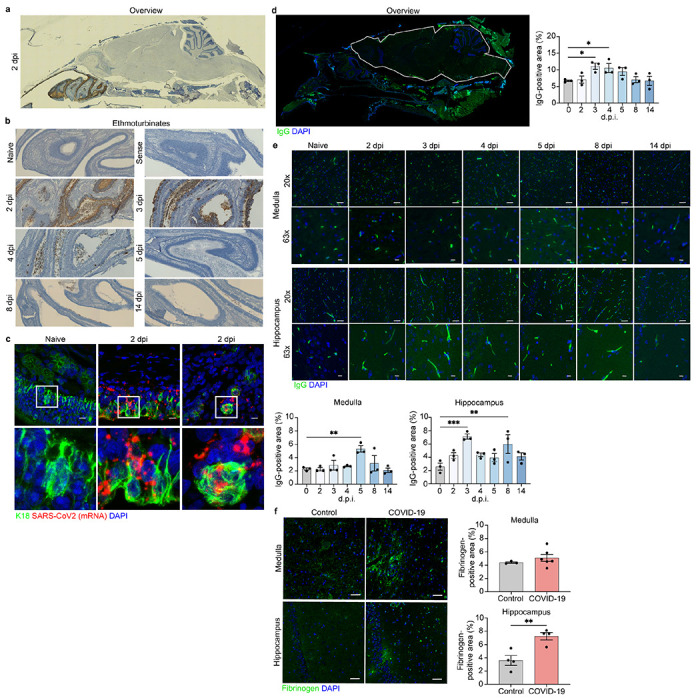
SARS-CoV2 infects hamster ONE and induces BBB disruption in hamsters and pa-tients with COVID-19. a. In situ hybridization for viral RNA at 2 dpi revealed SARS-CoV2 consistently targeted the ethmoturbinates of hamsters, with no infection of the CNS paren-chyma. b. Representative images of viral SARS-CoV2 mRNA in the hamster ethmoturbinates at naïve, 2, 3, 4, 5, 8, and 14 dpi. c. Co-localization of viral RNA (red) via in situ hybridization and immunodetection of K18+ sustenticular cells (green) of the ONE in naïve or SARS-CoV2-infected hamsters at 7 dpi. Nuclei counterstained with DAPI (blue), d. Representative image of blood-brain permeability in the hamster brain 2 dpi, showing staining for IgG (green) and DAPI (blue), followed by quantification of IgG intensity in the CNS parenchyma (white outline), e. Representative images of IgG detection (green) within hamster MO and hippocampi at naïve, 2, 3, 4, 5, 8, and 14 dpi, and nuclear stain, DAPI, (blue), followed by quantification of IgG intensity in their respective regions, f. Representative image of blood-brain permeability in the MO and hippocampus of control and COVID-19 patient tissue, depicting detection of fibrinogen (green) and DAPI (blue), followed by quantification of fibrinogen intensity. Data were pooled from at least two independent experiments. Scale bars, 50 μm (10x), 20 μm (20x) or 10 μm (63x). Data represent the mean ± s.e.m. and were analysed by two-way ANOVA or Student’s t-test.

The Golden Syrian hamster is naturally susceptible to SARS-CoV-2 infection. Intranasal inoculation results in mild-to-moderate disease with labored breathing, ruffled fur, weight loss, and hunched posture^5.^ To assess acute neuroinflammation following SARS-CoV-2 infection, 5-6-week-old male hamsters were infected intranasally with 2 x 10^5^ plaque forming units (PFU) of a fully infectious SARS-CoV-2 isolate (strain 2019-nCov/USA-WA1/20202). Whole heads of uninfected and infected hamsters were collected throughout the acute infectious period and at one week after viral clearance, which occurs in the lungs at 5-7 days post-infection (dpi)^18^. High levels of SARS-CoV-2 RNA was detected within the hamster ethmothurbinates at 2-4 days post infection (dpi), and completely cleared by 8 dpi (Fig. 1a,b). As previously reported^19^, viral RNA was detected only within K18^+^ sustentacular cells of the olfactory neuroepithelium (ONE) (Fig. 1c), which might impact olfactory sensory neuron (OSN) function via loss of calcium signaling^20^. SARS-CoV-2-infected sustentacular cells exhibited decreased expression of K18^+^ compared with uninfected ONE (Supplementary Video 1) and were found sloughed off into the nasal cavities of infected hamsters (Supplementary Video 2). Consistent with lack of OSN infection and neuroinvasion via the olfactory route, no viral RNA was detectable in the acute and recovered hamster olfactory bulb (OB), cortex, hippocampus, and medulla oblongata at any timepoint post-infection (Supplementary Fig. 1a).

Consistently with our hamster findings, we previously assessed 41 COVID-19 subjects who underwent autopsy and neuropathological exam, which we reported to show no viral RNA in brain tissue, as detected by RNAscope or RT-PCR^14^ (Supplementary Fig. 1b).

## SARS-CoV-2 infection is associated with widespread BBB disruption

Disruption of the BBB may occur during infection with respiratory viruses^21^. To evaluate effects of SARS-CoV-2 on BBB integrity in the hamster model, we assessed brain levels of extravasated serum IgG via immunohistochemistry. At 3-4 dpi there was a significant increase in IgG in the CNS parenchyma compared to naïve animals, which gradually decreased by 14 dpi (Fig. 1d). Further assessment of IgG extravasation revealed regional differences in BBB permeability following infection. While all regions examined, which include OB, cortex, hippocampus, and medulla oblongata, showed some degree of BBB disruption, the hippocampus suffered the most significant changes compare to naïve hamsters (Fig. 1e). The medulla oblongata exhibited little BBB disruption at early timepoints, but significant detection of IgG at 5 dpi (Fig. 1e). Similar persistent alterations in IgG immunoreactivity were observed in the OB and cortex, albeit to a lesser degree when compared to the hippocampus (Supplementary Fig. 2a).

We next examined BBB permeability in human COVID-19 brain tissue samples via detection of fibrinogen, a blood coagulation protein whose CNS deposition is implicated in a wide range of neurological disease and injuries associated with BBB disruption ^22^. In a subset of COVID-19 decedents (n=7) compared with age and sex-matched controls (Table 1) deceased from other infections (n=3) or cardiovascular disease (n=2), we observed increased fibrinogen in the hippocampus (Fig. 1f), and a smaller non-significant increase in medulla (Fig. 1f) and OB (Supplementary Fig. 2b). Together, these data suggest that SARS-CoV-2 infection may lead to region-specific alterations in human BBB integrity.

## SARS-CoV-2 infection is associated with aberrant expression of brain cytokines

Loss of BBB integrity may permit CNS entrance of cytokines or immune cells, which, in turn, may activate glial cells^23^. Given the observed increased BBB permeability in SARS-CoV-2 infected hamsters and humans, we examined microglia activation and cytokine expression by glia and neurons.

**Figure 2:**
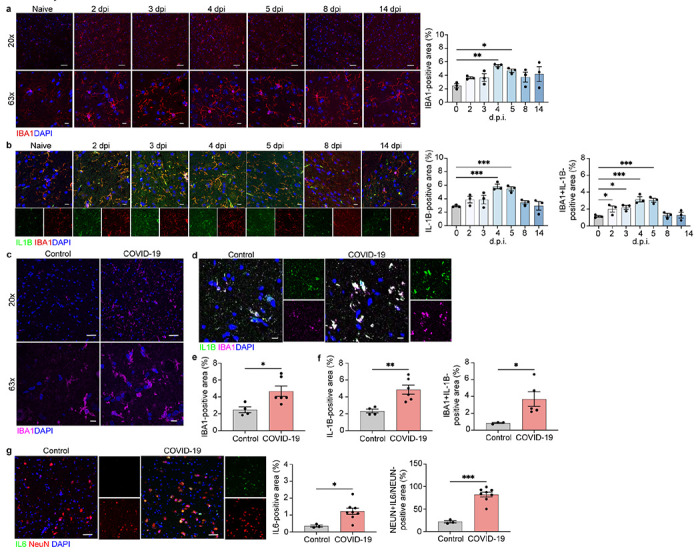
Microglia and neurons contribute to neuroinflammation in the ION of SARS-CoV-2-infected hamsters and of patients with COVID-19. a Representative image of IBA1 in the hamster ION at naïve, 2, 3, 4, 5, 8, and 14 dpi, showing staining for IBA1 (red) and DAPI (blue) at 20x and 63x and quantified for percent IBA1+ area. b Immunostaining for IL-1β and IBA1 in the hamster ION at naïve, 2, 3, 4, 5, 8, and 14 dpi, presented as microscopy with IBA1 (red), IL-1β (green) and DAPI (blue) and percent IL-1β+ area and IL-1β+IBA1+ area, normalized to total IL-1B+ area. c, e Representative image of IBA1 in control and COVID-19 patient ION, showing staining for IBA1 (magenta) and DAPI (blue) at 20x and 63x and quanti-fied for percent IBA1+ area. d, f Immunostaining for IL-1β and IBA1 in ION of control and COVID-19 patients, presented as microscopy with IBA1 (magenta), IL-1β (green) and DAPI (blue) and percent IL-1β+ area and IL-1β+IBA1+ area, normalized to total IBA1+ area. g. Im-munostaining for IL-6 and NeuN in ION of control and COVID-19 patients, presented as mi-croscopy with NeuN (red), IL-6 (green) and DAPI (blue) and percent IL-6+ area and IL-16+NeuN+ area, normalized to total NeuN+ area. Data were pooled from at least two independent experiments. Scale bars, 20 μm (20x) or 10 μm (63x). Data represent the mean ± s.e.m. and were analysed by two-way ANOVA or Student’s t-test.

Medulla oblongata from SARS-CoV-2-infected hamsters showed activated microglia within the inferior olivary nuclei (ION), displaying larger cell bodies and thicker processes than those detected in uninfected tissues, and increased levels of ionized calcium-binding adapter molecule 1 (IBA1) at 4 dpi, which remained elevated at 14 dpi (Fig. 2a). In vertebrates, ION are found in the medulla underneath the superior *olivary nucleus*, and coordinate signals from the spinal cord to the cerebellum, regulating motor coordination and learning via integration of glutamatergic synaptic inputs^24^.

Interleukin (IL)-1β and IL-6 are elevated in the cerebrospinal fluid of COVID-19 patients^25^, and IL-6 was reportedly elevated in CSF of children with acute encephalitis-like syndrome in the setting of infection with coronavirus-OC43^26^.

In the medulla ION, IBA1^+^ activated microglia of SARS-CoV-2-infected hamsters exhibited increased expression of IL-1β at 2-5 dpi, which returned to baseline by 8 dpi, compared to uninfected hamsters (Fig. 2b). Unfortunately, there are no commercially available antibodies to detect IL-6 in hamster tissues.

Analysis of COVID-19 patients revealed increased microglial activation, and IL-1β expression within the ION compared with control patient tissues (Fig. 2c–f). Elevated expression of IL-6 was detected in ION neurons, suggesting neuronal cytokine production (Fig. 2g), as previously reported^27^.

**Figure 3:**
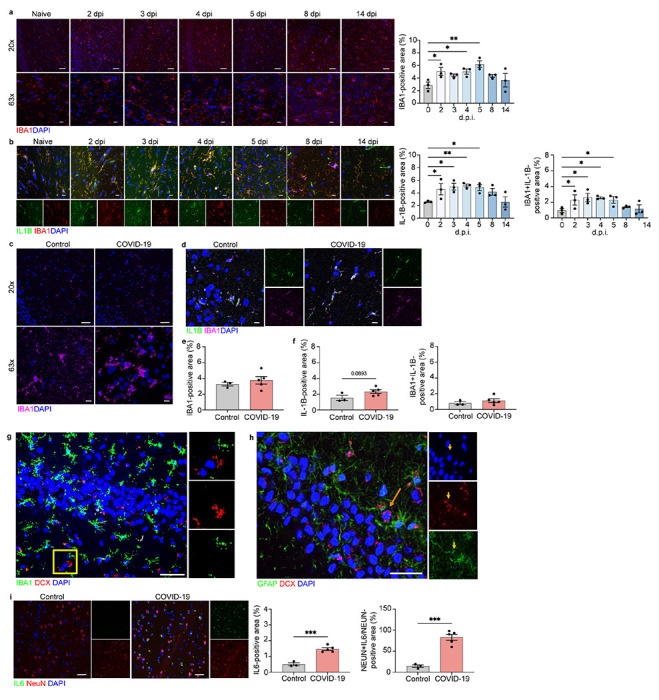
Microglia and neurons contribute to neuroinflammation in the hippocampi of viral-infected hamsters and COVID-19 patients, a Representative image of IBA1 in the hamster hippocampus at naïve, 2, 3, 4, 5, 8, and 14 dpi, showing staining for IBA1 (red) and DAPI (blue) at 20x and 63x and quantified for percent IBA1+ area. b Immunostaining for IL-1β and IBA1 in the hamster hippocampus at naïve, 2, 3, 4, 5, 8, and 14 dpi, presented as microscopy with IBA1 (red), IL-1β (green) and DAPI (blue) and percent IL-1β+ area and IL-1β+IBA1+ area, normalized to total IL-1B+ area. c, e Representative image of IBA1 in control and COVID-19 patient hippocampi, showing staining for IBA1 (magenta) and DAPI (blue) at 20x and 63x and quantified for percent IBA1+ area. d, f Immunostaining for IL-1β and IBA1 in hippocampi of control and COVID-19 patients, presented as microscopy with IBA1 (magenta), IL-1β (green) and DAPI (blue) and percent IL-1β+ area and IL-1β+IBA1+ area, normalized to total IBA1+ ar-ea. g. Representative images of IBA1 in the human adult hippocampus with high magnification images single channel. Sections stained with DAPI (blue), IBA1 (green), and DCX (red) in non-COVID-19 control. Scale bar, 25 μm. h Representative images of GFAP in the human adult hippocampus with high magnification images single channel. Sections stained with DAPI (blue), GFAP (green), and DCX (red) in non-COVID-19 control. The arrow points to a single DCX+/GFAP− neuron in the subgranular zone. Scale bar, 25 μm. i. Immunostaining for IL-6 and NeuN in hippocampi of control and COVID-19 patients, presented as microscopy with NeuN (red), IL-6 (green) and DAPI (blue) and percent IL-6+ area and IL-16+NeuN+ area, normalized to total NeuN+ area. Data were pooled from at least two independent experiments. Scale bars, 20 μm (20x) or 10 μm (63x). Data represent the mean ± s.e.m. and were ana-lysed by two-way ANOVA or Student’s t-test.

Hippocampal SARS-CoV-2-hamster tissue revealed a gradual increase in IBA1^+^ activated microglia versus uninfected hamsters, peaking at 5 dpi (Fig. 3a), and IL-1β levels increased at 2 dpi, peaking at 5 dpi before gradually decreasing to naïve levels by 14 dpi (Fig. 3b).

Hippocampal tissue from COVID-19 patients exhibited subtle changes in IBA1^+^ expression and microglial production of IL-1β compared with control patients (Fig. 3c–g). Similar to the medulla ION, we also observed neurons to be the main source of IL-6 in COVID-19 patient hippocampi compared to controls (Fig. 3i).

OB of both SARS-CoV-2-infected hamsters (2-5 dpi) and humans exhibited elevated expression of IL-1β within IBA1^+^ microglia compared with uninfected control tissues (Supplementary Fig. 4a,c). In hamster somatosensory cortex, co-localization of IL-1β within IBA1^+^ microglia was not significantly elevated (Supplementary Fig. 4b). In hamsters, OB and somatosensory cortex showed similar microglial activation, but this was not observed in OB of COVID-19 patients versus controls (Supplementary Fig. 3a–d). However, OB from COVID-19 patients exhibited elevated neuronal expression of IL-6 compared with controls (Supplementary Fig. 4d). IL-6 has been shown to have both pro- and anti-inflammatory roles^28^; further investigation of its role in COVID-19 is needed. Altogether, these data suggest neurons are important players in controlling neuroinflammation in humans affected by SARS-CoV-2.

We determined whether astrocytes contribute to neuroinflammation in the CNS of infected hamster and humans. Using a SOX9 antibody, we observed no persistent changes in astrocyte cell numbers in OB, ION, and hippocampus of infected hamsters compared to naïve animals (Supplementary Fig. 5). In COVID-19 human tissue samples, GFAP^+^ expression was increased in the medulla compared to controls (Supplementary Fig. 6a), and significant increases in IL-1β expression by GFAP^+^ astrocytes were observed in the OB of COVID-19 patients when compared to controls. No differences in astrocytes were observed between COVID-19 and control patients in the hippocampus and medulla oblongata (Fig. 3h, Supplementary Fig. 6a).

These data suggest that, while astrocytes may be involved in post-infection neuroinflammation in the OB, a region proximal to the location of viral replication, microglia and neurons appear to be the main players in more remote brain regions, like the medulla and hippocampus. Taken altogether, our findings show that, despite lack of viral neuroinvasion, SARS-CoV-2-infected individuals develop microglial activation and cytokine expression in brain regions associated with olfactory function, motor coordination, memory and learning, possibly inducing neuropsychiatric signs and symptoms.

## Loss of hippocampal neurogenesis in hamsters and humans infected with SARS-CoV-2

**Figure 4:**
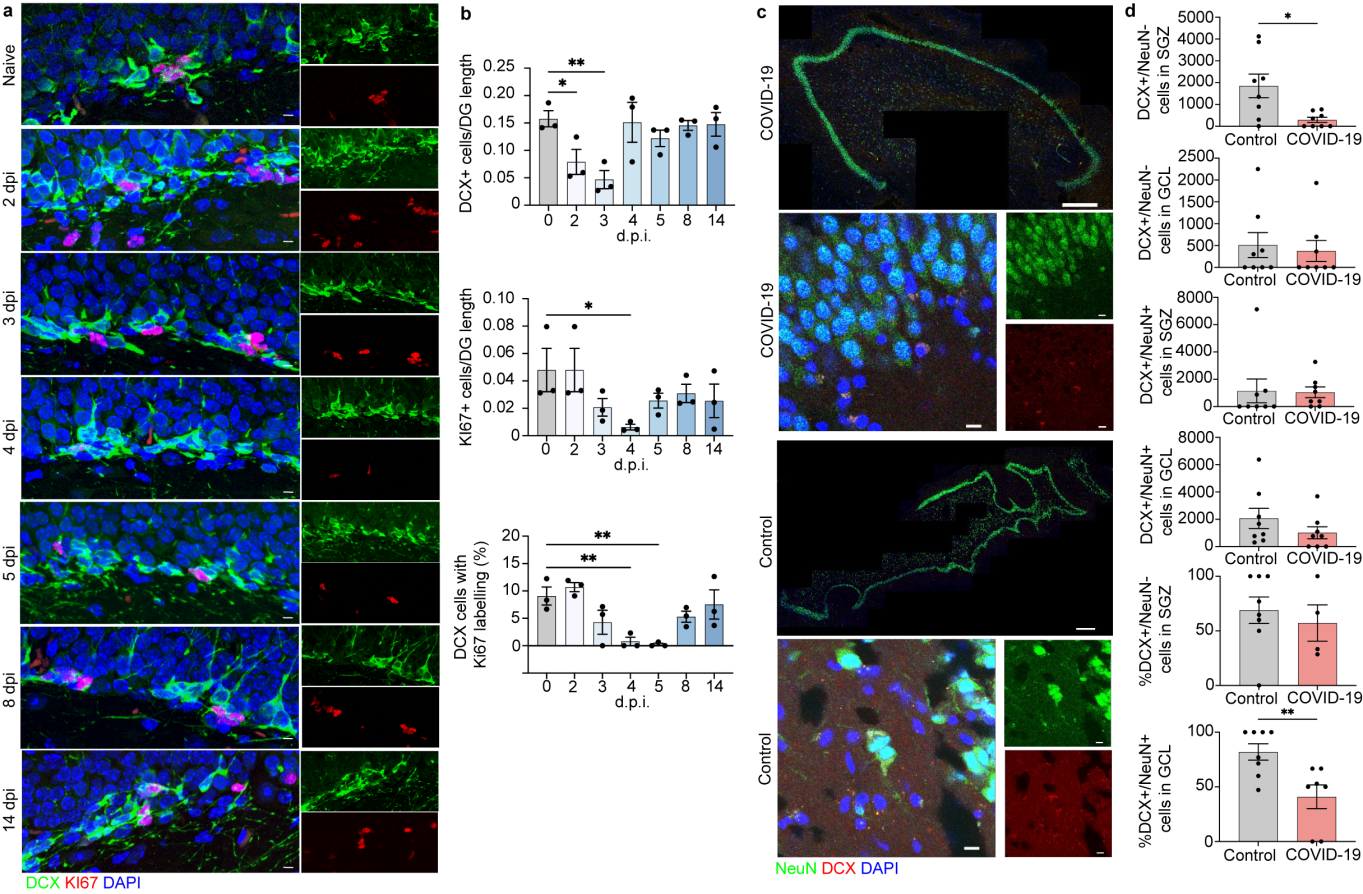
Neuroblast proliferation in the SARS-CoV2 infected hamster and doublecortin (DCX) positive cells and neurons in human hippocampus from COVID-19 patient and non-COVID-19 control. a, b Microscopy of the dentate gyrus of hamsters at naïve, 2, 3, 4, 5, 8, and 14 dpi, showing staining of Ki67 (red), neuroblast (green), and DAPI (blue), followed by quantification of percent Ki67+DCX+ cells, normalized to the total number of DCX+ cells. c. Quantification of percent DCX+Ki67+ area, normalized to total DCX+ area in the rostral migra-tory stream of hamsters at naïve, 2, 3, 4, 5, 8, and 14 dpi. Data were pooled from at least two independent experiments. Scale bars, 50 μm. Data represent the mean ± s.e.m. and were analysed by two-way ANOVA. c. Select images of whole hippocampus & high magnification images sections stained with DAPI (blue), NeuN (green), and DCX (red) from COVID-19 pa-tient and non-COVID-19 control. The granule cell layer (GCL), subgranular zone (SGZ), and molecular layer (ML) of the DG are visible, combined channels imaged at 20X; scale bar is 500 μm. High magnification images were captured at 63x, scale bar is 20 μm. d. In the SGZ, DCX+/NeuN− cells were fewer in COVID-19 patients vs controls (p = 0.026; t(7.794) = 2.731; Welch’s t test for non-stoichiometric data), with no group differences in DCX+/NeuN+ cell number (p = 0.189; Mann-Whitney). In the GCL, neither DCX+/NeuN− cell count (p = 0.846; Mann-Whitney), nor DCX+/NeuN+ cell count (p = 0.378; Mann-Whitney) differs between COVID-19 and control subjects. Percent of DCX+/NeuN− cells located in the SGZ vs the GCL did not differ between control and COVID-19 groups (p = 0.453; Mann-Whitney). Percent of DCX+/NeuN+ cells located in the GCL vs the SGZ was lower in COVID-19 patients vs control subjects (p = 0.009; Mann-Whitney).

The formation and consolidation of new memories occurs primarily within the hippocampus and relies on the integrity of a trisynaptic circuit between the entorhinal cortex, dentate gyrus (DG), and *Cornu Ammonis* (CA)^29^. Spatial learning, in particular, relies on the link between synapses within the CA3 region and rates of adult neurogenesis, which occurs via generation of new neurons from neural progenitor cells (NPCs) located in the subgranular zone (SGZ) of the DG^30^. Because adult neurogenesis may be affected by inflammation^31^, we hypothesized the cytokine surge following SARS-CoV-2 infection might affect adult neurogenesis. In rodents, adult hippocampal neurogenesis is a robust and well-established phenomenon^32,33^ and IL-1β reportedly inhibits neurogenesis during viral encephalitis, both acutely and after recovery^34^, while IL-6 represses neurogenesis through DNA demethylation/methylation^35^. Consistent with the detected increase in IL-1β and IL-6 in the hippocampus of SARS-CoV-2-infected hamsters, we observed a gradual decline in number of cells expressing Ki67, a marker of proliferation G0^36^, and doublecortin (DCX), a marker of neuroblasts and immature neurons^37^, as well as DCX^+^/Ki67^+^ cells, that were almost completely absent in the SGZ at 5 dpi (Fig. 4a,b). Cell numbers normalized to pre-infection levels after day 5. There was no change in DCX^+^/Ki67^+^ cell numbers in the rostral migratory stream in infected hamster compared to uninfected controls (Supplementary Fig. 7). These data suggest that effects of neuroinflammation and IL-1β on neurogenesis are specific to the DG.

To address the relevance of these findings in humans, we compared numbers of DCX^+^ neuroblast and DCX^+^/NeuN^+^ immature neurons in the SGZ and granule cell layer (GCL) of a subset of COVID-19 decedents (n=8), and age- and sex-matched non-COVID-19 controls (n=8, Table 1). Adult hippocampal neurogenesis has been shown to persist into adulthood in healthy humans^38,39^, with reports suggesting DCX^+^ immature neurons are present into the ninth decade of life^40^. Because DCX^+^ cells migrate from the SGZ into the GCL as they mature and start expressing NeuN^41^, DCX^+^/NeuN^−^ cells in the SGZ are more likely neuroblasts and DCX^+^/NeuN^+^ cells located in the GCL are more likely immature neurons^42^. We quantified these populations separately in the SGZ and GCL (Supplementary Fig. 8), and found fewer DCX^+^/NeuN^−^ neuroblasts in the SGZ of COVID-19 patients compared to non-COVID-19 controls (Fig. 4c,d). Conversely, DCX^+^/NeuN^−^ cells in the GCL and DCX^+^/NeuN^+^ immature neurons in SGZ and GCL were not fewer in COVID-19 versus control subjects (Fig. 4c,d). In mice neurogenesis takes approximately four weeks, and in monkeys it takes six months^43^, which is the time-frame these mammals carry their pregnancies. Thus, human neurogenesis might take nine months, and the more mature DCX^+^/NeuN^+^ cells, and DCX^+^/NeuN^−^ that have already migrated into the GCL, might have been generated weeks or months before the infection. On the other hand, cytokine surge and hypoxia appear to affect the more immature DCX^+^/NeuN^−^ cells located in the SGZ, possibly halting NPC differentiation into neuroblasts or reducing neuroblast survival. These hypotheses could be further tested in animal models where cell lineage can be traced, or in human brain via RNA velocity^44^.

We detected no significant effect of age on the number of DCX^+^/NeuN^+^ and DCX^+^/NeuN^−^ cells in SGZ and GCL in COVID-19 patients or non-COVID-19 controls (Supplementary Fig. 9), in line with previous findings on the persistence of adult hippocampal neurogenesis in older subjects with no chronic neuropsychiatric illness^38–40,45^.

## Limitations

Limitations of this study include the small sample sizes, and lack of reagents for detecting neural cell markers and additional inflammatory factors in hamsters. COVID-19 and non-COVID patients suffered hypoxia, which has been associated with activation of microglia in the absence of viral infection, and can also affect DCX^+^ cells^26^. Although most of our controls did not have a history of intubation, given their medical history of cardiovascular disease and infections, they likely experienced elevated cytokines and hypoxic damage. Hamsters develop milder disease without hypoxia, which is different from humans who died from COVID-19. Since hamsters exhibited similar CNS damage as humans, brain alterations are most likely the result of inflammation associated with COVID-19.

## Discussion

This study identifies neurobiological mechanisms of CNS damage in SARS-CoV-2 infection. In hamster and humans, BBB disruption, cytokine expression, activated microglia and loss of hippocampal neurogenesis, may contribute to neuronal dysfunction/loss, and ultimately to neurocognitive or psychiatric symptoms. The absence of these brain changes in non-COVID patients deceased from other infections or cardiovascular disease suggests effects of neuroinflammation and hypoxia in COVID-19 patients may be specific to SAR-CoV-2 infection.

Brain alterations were transient in hamsters, peaking after viral clearance in the nasal cavity. In humans, we do not know how long elevated inflammatory markers persist in subjects who survive the disease. The persistence of neuropsychiatric symptoms in long-COVID cases suggests that neuronal damage may be prolonged. Brain imaging studies investigating inflammation markers in post-COVID patient are warranted. Studies using positron emission tomography (PET) radiotracers for the brain translocator protein (TSPO)^46^ located on microglia and astroglia, and transcranial near-infrared spectroscopy (NIRS) to assess mitochondrial function^47,48^, may reveal useful for gathering data on indices of brain inflammation levels in post-COVID patients.

In hamsters and humans, BBB disruption and cytokine expression by microglia, astrocytes, and neurons occurred in a region-specific fashion and were not associated with viral neuroinvasion^14^. Therefore, cytokines appear to be the main mediator of BBB disruption and cellular damage. NPC IL-1β receptors have been implicated in reducing neurogenesis in murine models of flavivirus encephalitis^34,50^. These viruses, however, lead to significant CNS infiltration of mononuclear cells, which has not been observed in studies of COVID-19.

Fewer DCX^+^ neuroblasts in the SGZ of COVID-19 patients compared with non-COVID-19 controls suggests either decreased NPC maturation or increased neuroblast death. DCX expression has been hypothesized to occur in mature granule neurons that might be de-differentiating in pathological conditions^51^. However, this kind of cells would be more likely DCX^+^/NeuN^+^ cell located in the GCL, which were unaffected. The time-course of human pathological findings remains unknown, and if similar to hamsters, one would expect that after the initial cytokine surge, neurogenesis might recover, as cognitive symptoms and anosmia subside in many patients. Nevertheless, long-COVID symptoms have been widely reported^12^. There is the possibility that, in some individuals, the neurogenic niche might not have enough multipotent progenitors for neurogenesis to resume after this insult, as the multipotent progenitor pool of SOX2^+^ cells is smaller in aging humans^38^. If this was the case, some patients might not be able to recover, and COVID-19 may result in chronic neuropsychiatric symptoms, as observed in several clinical studies^2,8,13^.

Given the likely predominant role of neuroinflammation in the mechanism of brain damage in COVID-19, anti-IL-6 and anti-IL-1β therapies, currently under investigation^52,53^, could be useful in limiting a prolonged cytokine storm, possibly preventing motor, cognitive, neurovegetative and emotional dysfunctions.

## Supplementary Material

Supplement 1

Supplement 2

Supplement 3

Supplement 4

Supplement 5

Supplement 6

Supplement 7

Supplement 8

Supplement 9

Supplement 10

Supplement 11

Supplement 12

Supplement 13

Supplement 14

## Figures and Tables

**Table 1.** Patient Demographics and Clinical Characteristics for COVID-19 Cases and non-COVID-19 Controls

	Cases (n=17)		Controls (n=13)	

	Mean	SD	Mean	SD
Age	68	15	66	11
Post-mortem interval (hours)	20	24	20	9
Days of symptoms prior to hospitalization	6	5	NA	
Hospitalization days (a)	19	18	3.3	8.4
	N	( % )	N	( % )

Sex				
Female	8	( 47 )	5	( 38 )
Male	9	( 53 )	8	( 63 )
Ethnicity				
Caucasian	6	( 35 )	11	( 85 )
Declined to report	3	( 38 )	0	( 0 )
Psychiatric diagnosis				
None	14	( 82 )	13	( 100 )
Anxiety/depression	3	( 18 )	0	( 0 )
Pre-existing cognitive impairment	3	( 18 )	0	( 0 )
Neurological/psychiatric symptoms at hospital presentation	7	( 41 )	2	( 15 )
Steroids during hospitalization	5 / 8	( 63 )	0	( 0 )
Tocilizumab/remdesivir during hospitalization	3 / 8	( 36 )	0	( 0 )
Other immunosuppressive medications	2 / 8	( 25 )	0	( 0 )
Blood inflammatory markers during hospitalization (ESR, CRP, LDH, ferritin)				
Elevated	11	( 65 )	0	( 0 )
Not measured	6	( 35 )	13	( 100 )
Intubated	9	( 53 )	1	( 8 )
Stroke at death	5	( 29 )	0	( 0 )
Primary cause of death				
Cardiovascular	4	( 24 )	6	( 46 )
Infection (ileus, broncopneumonia, bacterial endocarditis, sepsis)	11	( 65 )	5	( 39 )
Uremia	0	( 0 )	1	( 8 )
Stroke/dissection	2	( 11 )	0	( 0 )
Accidental	0	( 0 )	1	( 8 )
Positive brain toxicology (b)	10	( 59 )	1	( 13 )
Positive blood toxicology (c)	NT		2	( 25 )
Tobacco use disorder	1	( 6 )	1	( 13 )

Note:

(a) Agonal hospitalization time was available for 5 of the controls; average was 5 hours.

(b) Cases: 4 patients intubated with propofol, fentanyl, benzodiazepine, dexmedetomidine. One patient on comfort care with fentanyl and benzodiazepine. Controls: Lidocaine measured in one post-mortem brain.

(c) One control had lidocaine, N-acetylprocainamide, and procainamide, another had caffeine and lidocaine.

NT = Not tested, due to limited resources during initial months of pandemic. NA = not available.

## Data Availability

The data from this study are tabulated in the main paper and supplementary materials. All reagents are available from authors under a material transfer agreement with Washington University or Columbia University Medical Center.
