# Supplementary material for: COVID-19 induces neuroinflammation and loss of hippocampal neurogenesis
Source: Res Sq. 2021 Oct 29:rs.3.rs-1031824. Preprint. [Version 1] doi: 10.21203/rs.3.rs-1031824/v1 (PMC8562542; doi:10.21203/rs.3.rs-1031824/v1)
Supplement: Supplement 1 [file 06103e211330eea6566c47f2.docx]

**Supplementary Materials**

**Methods**

**Hamster Studies**

**Golden Syrian Hamsters:** Five to six-week-old male hamsters were used for all experiments. Golden Syrian hamsters were obtained from Charles River Laboratories. All experiments followed guidelines approved by the Washington University School of Medicine Animals Safety Committee.

**Hamster model of SARS-CoV2- infection:** SARS-CoV2 (2019-nCoV/USA-WA/2020 strain) was propagated on MA-104 monkey kidney cells. Animals were administered 2.5 x 10^5^ PFU of SARS-CoV-2 by the intranasal route in a final volume of 100 µL. Inoculations were performed under anaesthesia that was induced and maintained with 5% isoflurane. All hamsters were monitored for body weight until humanely euthanized.

**Hamster tissue immunohistochemistry (IHC):** Hamster brains still encased in the skull were fixed for seven days in 10% formalin and then decalcified in 0.5 M EDTA for seven days. Brains were then cryopreserved in 30% sucrose (three exchanges for 24h). Prior tissue sectioning (10 µm), samples were frozen in OCT compound (Fischer). Tissue sections were boiled in 10 mM sodium citrate buffer, 0.05% Tween 20, pH 6.0 for 30 min and then washed with PBS. Tissue sections were permeabilized with 0.1–0.3% Triton X-100 (Sigma-Aldrich), followed by 1 h of blocking with 5% normal goat serum (Sigma-Aldrich) at room temperature. Slides were then incubated in primary antibody overnight at 4 °C. The following day, slides were incubated in secondary antibodies at room temperature for 1 h, and nuclei were counterstained with 4,6-diamidino-2-phenylindole (DAPI; Invitrogen). Slides were covered slipped with ProLong Gold Antifade (Thermo Fisher). Images were acquired on a Zeiss LSM 880 confocal laser scanning microscope with Zeiss software. Immunofluorescent signals were quantified using the software ImageJ. Cells in the identified region of interest were counted in an unbiased fashion using the provided ImageJ plugin, Cell Counter.

**Human Studies**

**COVID-19 Subjects Tissue Collection:** COVID-19 brain tissues were provided by the Columbia University BioBank at Columbia University Irving Medical Center and the New York Presbyterian Hospital. Consent for autopsy was obtained from patient surrogates through standardized consenting procedures via telephone, given that no visitors were allowed in hospital during the study time. We examined subjects of all consecutive autopsies performed between March through June, 2020, meeting the Center for Disease Control and Prevention case definition for definitive COVID-19 infection (https://wwwn.cdc.gov/nndss/conditions/coronavirus-disease-2019-covid-19/casedefinition/2020/). Individuals included in this study had been treated at the Columbia University Irving Medical Center.

**COVID-19 Subjects Autopsy procedures:** All COVID-19 cases autopsies were conducted in a negative pressure room. The brain with the attached uppermost spinal cord was removed after the general autopsy using a bone saw with a vacuum attachment to minimize personnel exposure to bone dust. Sections (up to approximately 3 cm in greatest dimension) from the olfactory bulb/gyrus rectus, superior frontal gyrus, mesial temporal lobe with the anterior hippocampus and amygdala, cerebellum and medulla oblongata were placed in clean plastic bags, sealed, marked, and frozen on dry ice and stored at -80° C in the Columbia University BioBank (CUB) laboratory dedicated to storing and processing COVID-19 autopsy tissues. After removing tissue for freezing, each brain was fixed in 10% buffered formalin for 10 days. After brain removal, parallel longitudinal cuts were made in the medial anterior cranial fossa to circumferentially excise the cribriform plate of the ethmoid bones along with underlying olfactory and nasal epithelium. A small piece of nasal epithelial tissue was sampled for qRTPCR, and the remainder divided for freezing and for fixation in 10% formalin.

**COVID-19 Subjects Clinical Characteristics:**

Clinical data including demographics, clinical, laboratory, radiographic, and treatment data were obtained retrospectively from electronic medical record (EMR) review. Presenting symptoms were considered symptoms present in admission documentation. Admission documentation was reviewed manually. The hospital EMR was used to search the neuropsychiatric search terms listed in Table 1 (Baseline Characteristics) as well as other common hospital terms (“altered mental status, AMS, disoriented, disorientation”). Documents with mentions of these terms were then reviewed for appropriateness of inclusion. In addition, notes after admission were reviewed for symptoms described by the patients’ relations to have occurred prior to presentation. All laboratory tests, neuroradiology assessments, and interventions were performed at the discretion of the treating physicians. Inclusion Criteria: All post-mortem patients >18 years of age undergoing autopsy analysis for COVID-19. Exclusion Criteria: All individuals <18 years of age and those individuals who do not undergo autopsy.

**Control Subjects Tissue Collection:** Brain tissue was obtained from the Brain Collection of the New York State Psychiatric Institute at Columbia University, which includes brain samples from the Republic of Macedonia. Brain tissue collection was conducted with IRB approval and consent obtained from all informants.

Brain tissue was also provided by Robert Schmidt at Washington School of Medicine. Brain tissue collection was conducted with IRB approval and consent obtained from all informants (Table 1).

**Control Subject Tissue Collection:** At autopsy, 2 cm-thick coronal blocks of the right hemisphere were flash-frozen in liquid Freon (−20°C) and stored at −80°C. Tissue samples were fixed in formalin for neuropathological examination. Brain pH determination^54^ and toxicology were performed on cerebellar samples and blood. Over 30 drugs were screened for and quantified including alprazolam, amitriptyline, amphetamine, buspirone, clomipramine, citalopram, clozapine, cocaine, diazepam, fluoxetine, fluvoxamine, haloperidol, imipramine, methadone, nortriptyline, paroxetine, olanzapine, and sertraline.

**Control Subject Characteristics and Matching Procedure:** Subjects were screened for psychiatric illness using our validated psychological autopsy for DSM diagnoses^55^. As part of the clinical evaluation, GAS score was measured. Subjects included in the study had no neurological or psychiatric diagnoses, no pathological or traumatic condition affecting the brain, clear toxicology reports, and were free of psychiatric treatments and cognitive impairment. Exclusion criteria were: any neuropsychiatric diagnosis, positive toxicology for psychoactive drugs or alcohol, alcoholism-associated liver changes, suicide attempt history, intellectual disability, AIDS, positive neuropathology, undetermined death, prescription of psychotropic medications in the last three months prior to death, long agonal state, and any chronic disease. Subjects with the presence of diseases affecting the brain were excluded using clinical data, psychological autopsy interviews and neuropathological exams.

**Human Brain Tissue Processing:** Brain regions of interest were dissected from coronal blocks, fixed, cryoprotected in 30% sucrose, sectioned at 50 μm on a sliding microtome (Microm HM440E) and stored in 40-wells boxes at −20°C in cryoprotectant (30% ethylene glycol in 0.1M PBS). One section every 500 microns was set aside for Nissl staining during the sectioning procedure. Nissl-stained sections were later used for anatomical alignment along the DG rostro-caudal axis of sections processed for immunocytochemistry.

**Human Brain Tissue Immunofluorescence:** Double immunofluorescence was performed to visualize and quantify markers colocalization. Brain tissue sections were washed in 0.05 M Phosphate Buffer Saline (3x10 min) then incubated in 1% Sodium Borohydride (15min) in 0.05 M PBS, followed by another wash in 0.05 M PBS (3x10’) and an incubation in 3% H_2_O_2_ in 0.05 M PBS. After a wash (3x10min. in 0.05M PBS), sections were blocked in blocking solution (0.3% Triton X-100 (Sigma-Aldrich), 20% normal goat serum (Vector Laboratories), 2% bovine serum albumin (Santa Cruz)) for 2 hours at room temperature. Subsequently, sections were incubated overnight at 4C in a 1:4 blocking solution with the following primary antibodies: 1:500 guinea pig anti-DCX IgG (Sigma-Aldrich) and 1:500 rabbit anti-NeuN IgG (Cell Signaling). The next day, tissue sections were incubated with secondary antibodies; 1:1000 594-AffiniPure goat anti-guinea pig IgG (Jackson ImmunoResearch) and 1:1000 488-AffiniPure goat anti-rabbit IgG (Jackson ImmunoResearch) and incubated overnight in the dark at 4C. Sections were washed in PBS(3X10min), incubated with DAPI (4’,6-diamidino-2-phenylindole) and treated with 0.1% Sudan Black in 70% ethanol for 5 minutes (to reduce innate auto-fluorescence of human brain), then finally washed 5x10’ in PBS, mounted, dried for a few hours and coverslipped with 90% glycerol.

**Human Brain Tissue Confocal Scanning:** To quantify co-localization of cell markers, image stacks of the whole DG processed for immunohistofluorescence were obtained using a confocal scanning microscope (Leica TCS SP8 2-Photon, Leica Microsystems Inc.), and then processed for Stereology using Stereoinvestigator software (MBF, Inc.). The Leica TCS SP8 2-Photon microscope is equipped with three simultaneous PMT detectors. Fluorescence from the different fluorophores was detected in the following way: 1) 488-AffiniPure was excited at 488 nm and detected at 505-550 nm, 2) 594-AffiniPure was excited at 552 nm and detected at 600-650 nm, and 3) DAPI was excited at 405 nm and detected at 415-485 nm. For 488- and 552-nm excitation, the beam path included a TD 488/552/638 beamsplitter, while for 405 nm excitation a substrate beamsplitter was used. Tiled z stacks of the entire hippocampus area were obtained by first imaging conjugated fields of view via an automated stage in conjunction with the Stage Overview function of the LAS X software (Leica Application Suite X 3.1.1.15751), and then by stitching the images with the merging algorithm provided with the software. To select the area of the hippocampus on each section, the Stage Overview function was utilized. This function allowed for the fast acquisition of a low-resolution preview scan of the entire tissue section containing the hippocampus, using a 10x objective, and next for selecting the regions of interest surrounding the dentate gyrus to be included in the final tiled z stacks. Three (3) 50um thick tissue section from each individual were sampled around the mid-anterior body of the hippocampus, Image stacks from each tissue section assayed for DCX/NeuN, were acquired applying the same parameters to the Leica TCS SP8 2 photon microscope (Leica Microsystems Inc.). All z stacks were imaged with a dry Leica 20X objective (NA 0.70, working distance 0.5 mm), with a field of view of 553.6 3 553.6 mm, a pixel size of 0.54 3 0.54 mm, optical sectioning of 2.36 mm, and a z step of 1 mm. Depending on the thickness of each tissue section, the final z stack was determined to be 13 ± 2 mm. Image stacks were then imported in our MicroBrightfield system (MBF Bioscience, Williston, VT) to perform unbiased stereology.

**Human Brain Tissue Stereology:** To estimate total cell counts, we adapted an optical *disector* with fractionator method, an unbiased stereological approach allowing to estimate the total number of cells in the region of interest (Stereo Investigator software, MBF Bioscience, Williston, VT). Using the Stereo Investigator Software, first, the dentate gyrus boundaries were defined tracing an outline including the granular cell layer, subgranular zone, and molecular layer. Then, the software systematically generates sample sites based on the user defined parameters for counting frame width and height (80μm), sampling grid in the X and Y plane (200μm), sampling grid area (4000 μm), and *disector* height in z plane (approximately 7μm). Mean section thickness was based on the thickness at which the tissue was sectioned at the microtome (50μm). The user defined section thickness (11-19μm) was the depth of the z plane of the section after undergoing IF and defining the safety guard zones (3μm) at the top and bottom of the z stack.

To estimate the total number of cells, $N$, Stereo Investigator software uses the following formula:

$$N= \sum Q^{-}\times\frac{t}{h}\times\frac{1}{asf}\times\frac{1}{ssf}$$

where $\sum Q^{-}$ is the total number of cells counted in the sampling sites, $t$ is the mean section thickness, $h$ is the height of the optical disector adjusted for the guard zones above and below, $asf$ is the area sampling factor, and $ssf$ is the section sampling fraction. We analysed two sections of the anterior hippocampus per subject, we computed the estimated cell number and the region volume, from which cell density was calculated as: cell number/cubic mm of tissue.

**Human Brain Tissue Statistical Analysis:** Data analysis was performed using SPSS (version 24 for Mac). COVID patients were paired with demographic matched controls to control for confounding variables like age. Reported N values represent the estimated cell counts by stereology in the dentate gyrus. Significance level was set at p < 0.05.

**Supplemental Figures and Videos**

**Supplementary Figure 1: Blood-brain breakdown occurs through the CNS of SARS-CoV2-infected hamster and human COVID-19 patients.** **a** In situ hybridization for SARS-CoV2 mRNA in the hamster OB, cortex, hippocampus, and MO. **b** Similar evaluation for SARS-CoV-2 RNA in the OB, cortex, or medulla of the human COVID 19 patients. Scale bars, 10 μm (C).

**Supplementary Figure 2: a** Representative images of IgG detection (green) within hamster OB and cortices in uninfected and SARS-CoV-2-infected hamsters at 2, 3, 4, 5, 8, and 14 dpi, and nuclear stain, DAPI, (blue), followed by quantification of IgG intensity in their respective regions. **b** Representative image of fibrinogen (green) detection in the OB and cortices of control and COVID-19 patient tissues, and DAPI (blue), followed by quantification of fibrinogen intensity. Data were pooled from at least two independent experiments. Scale bars, 50 μm (A) or 20 μm (B). Data represent the mean ± s.e.m. and were analyzed by two-way ANOVA.

**Supplementary Figure 3: Microglia activation occurs in the OB and cortices of SARS-CoV-2-infected hamsters and in OB of COVID-19 patients. a, b** Representative images of IBA1 (red) in the uninfected and SARS-CoV-2-infected hamster OB (A), and cortices (B) at, 2, 3, 4, 5, 8, and 14 dpi, and DAPI (blue) at 20x and 63x and quantified for percent IBA1^+^ area. **c** Representative images of IBA1 (magenta) in control and COVID-19 patient olfactory bulb, and DAPI (blue) at 20x and 63x and quantified for percent IBA1^+^ area. Data were pooled from at least two independent experiments. Scale bars, 20 μm (20x) or 10 μm (63x). Data represent the mean ± s.e.m. and were analyzed by two-way ANOVA.

**Supplemental Figure 4: Microglia production of IL-β is observed throughout the OB and cortices of SARS-CoV-2-infected hamsters and in OB of COVID-19 patients. a, b** Immunostaining for IL-1β and IBA1 in the uninfected and SARS-CoV-2-infected hamster OB (A), and cortex (B) at 2, 3, 4, 5, 8, and 14 dpi, presented as microscopy with IBA1 (red), IL-1β (green) and DAPI (blue) and percent IL-1β^+^ area and IL-1β^+^IBA1^+^ area, normalized to total IBA1^+^ area. **c** Immunostaining for IL-1β and IBA1 in OB control and COVID-19 patients, presented as microscopy with IBA1 (magenta), IL-1β (green) and DAPI (blue) and percent IL-1β^+^ area and IL-1β^+^IBA1^+^ area, normalized to total IBA1^+^ area. Data were pooled from at least two independent experiments. Scale bars, 20 μm. Data represent the mean ± s.e.m. and were analyzed by two-way ANOVA.

**Supplemental Figure 5: Astrocyte cell numbers do not increase in the CNS of hamsters infected with SARS-CoV2. a** Representative image of SOX9 in the hamster olfactory bulb (A), cortex (B), hippocampus (C) and medulla (D) at naïve, 2, 3, 4, 5, 8, and 14 dpi, showing staining for SOX9 (red) and DAPI (blue) and quantified for number of SOX9^+^ cells. Data were pooled from at least two independent experiments. Scale bars, 20 μm. Data represent the mean ± s.e.m. and were analyzed by two-way ANOVA.

**Supplemental Figure 6: Astrocytic production of IL-1β inconsistently contributes to neuroinflammation in the CNS of COVID-19 patients. a** Immunostaining for IL-1β and GFAP in hippocampi, olfactory bulbs, and medulla of control and COVID-19 patients, presented as microscopy with GFAP (red), IL-1β (green) and DAPI (blue) and percent IL-6^+^ area and IL-16^+^NeuN^+^ area, normalized to total GFAP^+^ area. Data were pooled from at least two independent experiments. Scale bars, 20 μm. Data represent the mean ± s.e.m. and were analyzed by two-way ANOVA.

**Supplemental Figure 7: Neuroblast proliferation is unaffected in the rostral migratory stream of SARS-CoV-2-infected hamsters.** Quantifcation of percent DCX^+^Ki67^+^ area, normalized to total DCX^+^ area in the rostral migratory stream of hamsters at naïve, 2, 3, 4, 5, 8, and 14 dpi. Data were pooled from at least two independent experiments. Scale bars, 50 μm. Data represent the mean ± s.e.m. and were analyzed by two-way ANOVA.

**Supplementary Figure 8: Unbiased stereological count of doublecortin (DCX) and neuronal nuclear marker (NeuN) expressing cells in human adult hippocampus.** To quantify co-expression of DCX and NeuN in neurons, image stacks of the whole DG, processed for immunohistofluorescence, were obtained using a confocal scanning microscope (Leica TCS SP8 2-Photon, Leica Microsystems Inc.) then processed by unbiased Stereology using Stereoinvestigator software (MBF, Inc.). **a.** Anatomical delineation of the Granular Cell Layer (GCL) + Subgranular cell layer (SGL). **b.** 3D counting frame for unbiased sampling, White dotted line squares indicate sampling frames while smaller red and green squares indicate the counting frame (**c**). **d.** Example of a counted hippocampus, Yellow crosses indicate counted cells that were DCX^+^/NeuN^+^. Pink triangles indicate cells that were DCX^+^/NeuN^-^.

**Supplementary Figure 9:** **Correlations between DCX+ cell number and age by cell maturity and location.** **a**. In the SGZ, stereologically estimated DCX+/NeuN- cell number did not correlate significantly with age in control subjects (p = 0.139; r = -.571) or COVID-19 patients (p = 0.779; r = .132). **b**. Likewise, in the SGZ, DCX+/NeuN+ cell number did not correlate significantly with age in control subjects (p = 0.342; r = -.388) or COVID-19 patients (p = 0.438; r = -.353). **c**. In the GCL, DCX+/NeuN- cell number did not correlate significantly with age in control subjects (p = 0.159; r = -.549) or COVID-19 patients (p = 0.187; r = .564). **d**. Likewise, in the GCL, DCX+/NeuN+ cell number did not correlate significantly with age in control subjects (p = 0.149; r = -.561) or COVID-19 patients (p = 0.797; r = .121).

**Supplementary Video 1: 3D reconstruction from confocal Z-stack images of immunostaining of SARS-CoV-2 viral mRNA infection of naïve hamster K18+ sustentacular.** This video shows a 3D reconstruction from confocal Z-stack images of immunostaining of SARS-CoV-2 viral mRNA, and the sustentacular marker, K18, in the olfactory neuroepithelium of a naïve hamster.

**Supplementary Video 2: 3D reconstruction from confocal Z-stack images of immunostaining of SARS-CoV-2 viral mRNA infection of hamster K18+ sustentacular at 2 dpi.** This video shows a 3D reconstruction from confocal Z-stack images of immunostaining of SARS-CoV-2 viral mRNA, and the sustentacular marker, K18, in the olfactory neuroepithelium of a SARS-CoV-2-infected hamster 2 dpi.

**Supplementary Video 3: Video from Z-stack images of neuroblast immunostaining in the GCL of a COVID-19 patient.** Video of z-stack of DCX+/NeuN+ cell in granule cell layer (GCL) from COVID-19 patient. Imaged at 63X. Z-stack includes 21 images 0.4 μm apart.

**Supplementary Video 4: Video from Z-stack images of neuroblast immunostaining in the GCL and SGZ of a COVID-19 patient.** Video of z-stack of DCX+/NeuN+ cell in granule cell layer (GCL) and subgranular zone (SGZ) of human dentate gyrus from COVID-19 patient. Imaged at 63X. Z-stack includes 21 images 0.4 μm apart. Images are 332 by 176 μm. Red arrows indicate DCX+/NeuN+ cell in GCL. White arrows indicate DCX+/NeuN+ cell in SGZ.
