# Supplementary figures and images for: COVID-19 induces neuroinflammation and loss of hippocampal neurogenesis
Source: Res Sq. 2021 Oct 29:rs.3.rs-1031824. Preprint. [Version 1] doi: 10.21203/rs.3.rs-1031824/v1 (PMC8562542; doi:10.21203/rs.3.rs-1031824/v1)

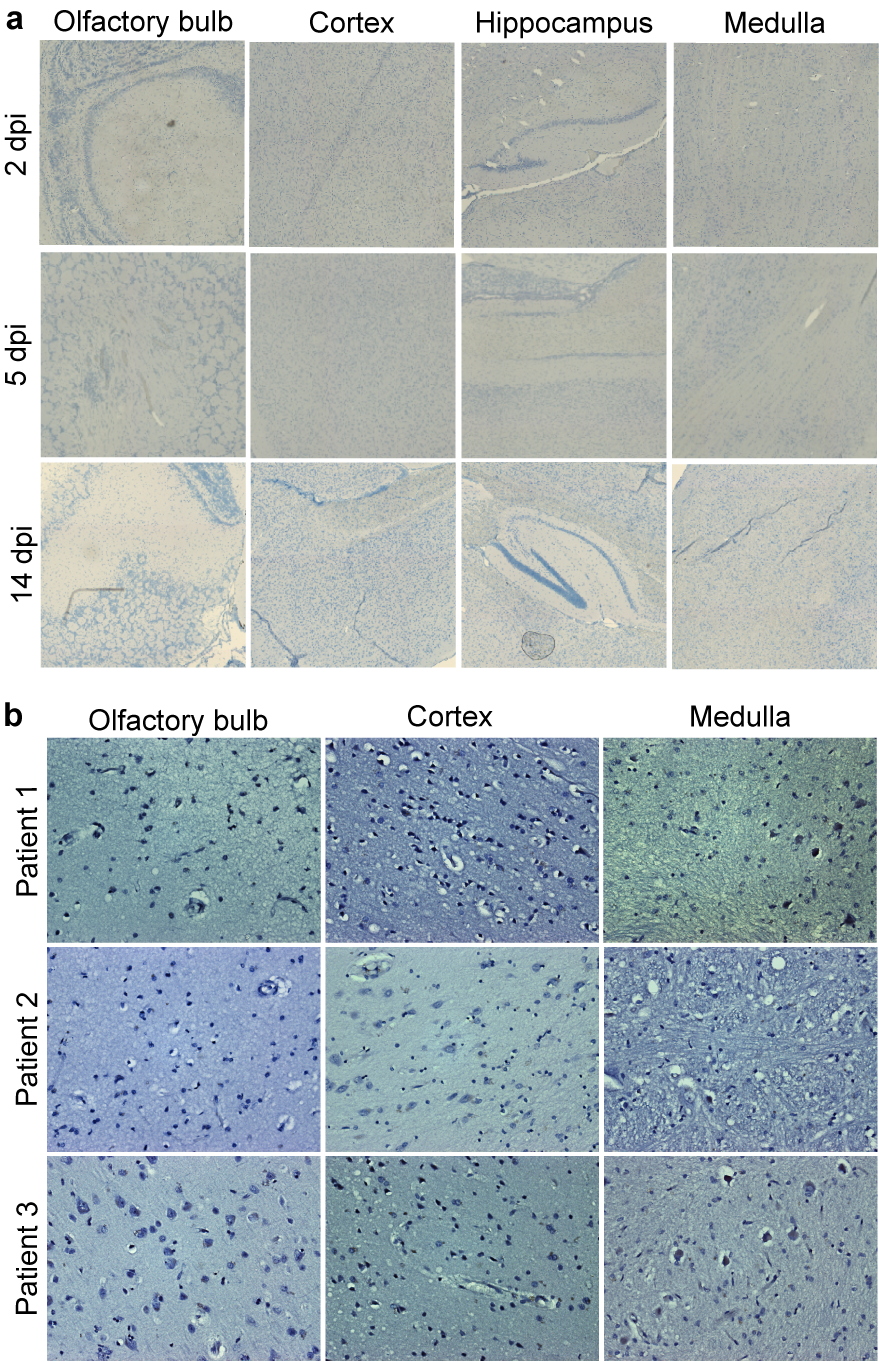

Supplement: Supplement 6 [file d7dcade9b9b74387a7d3824b.tif]

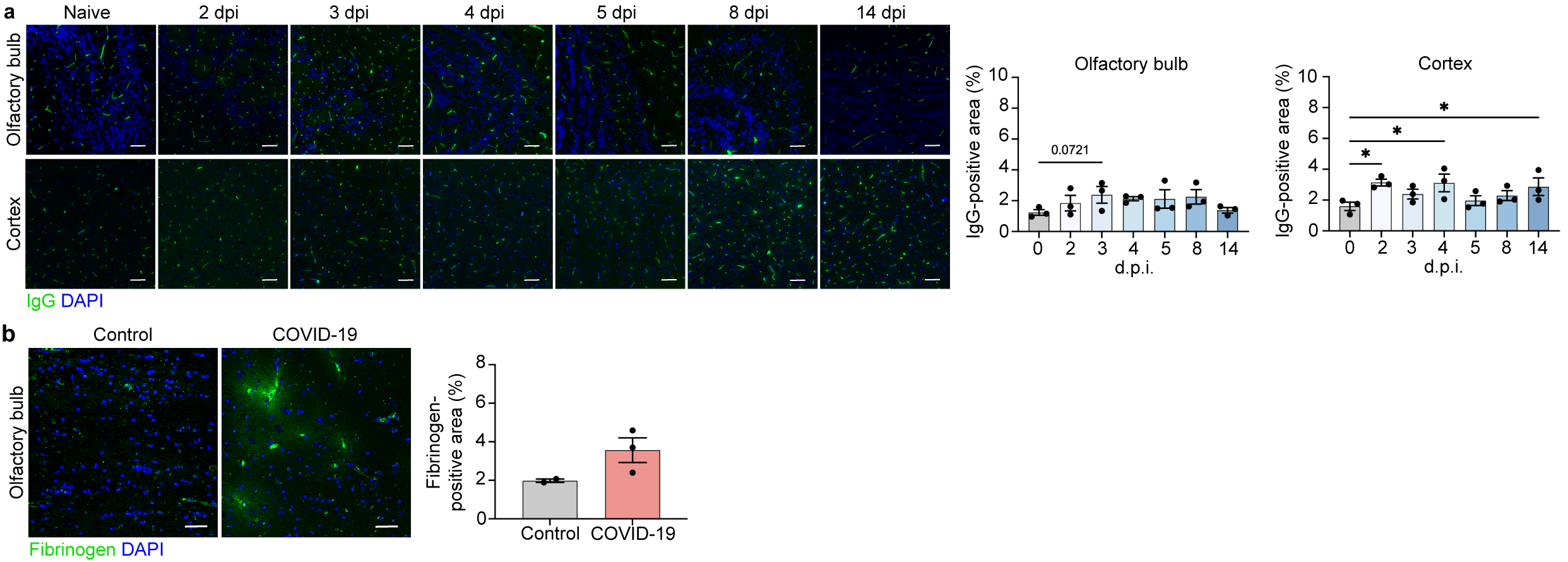

Supplement: Supplement 7 [file 589fda7cb3dfbfa0516a6912.tif]

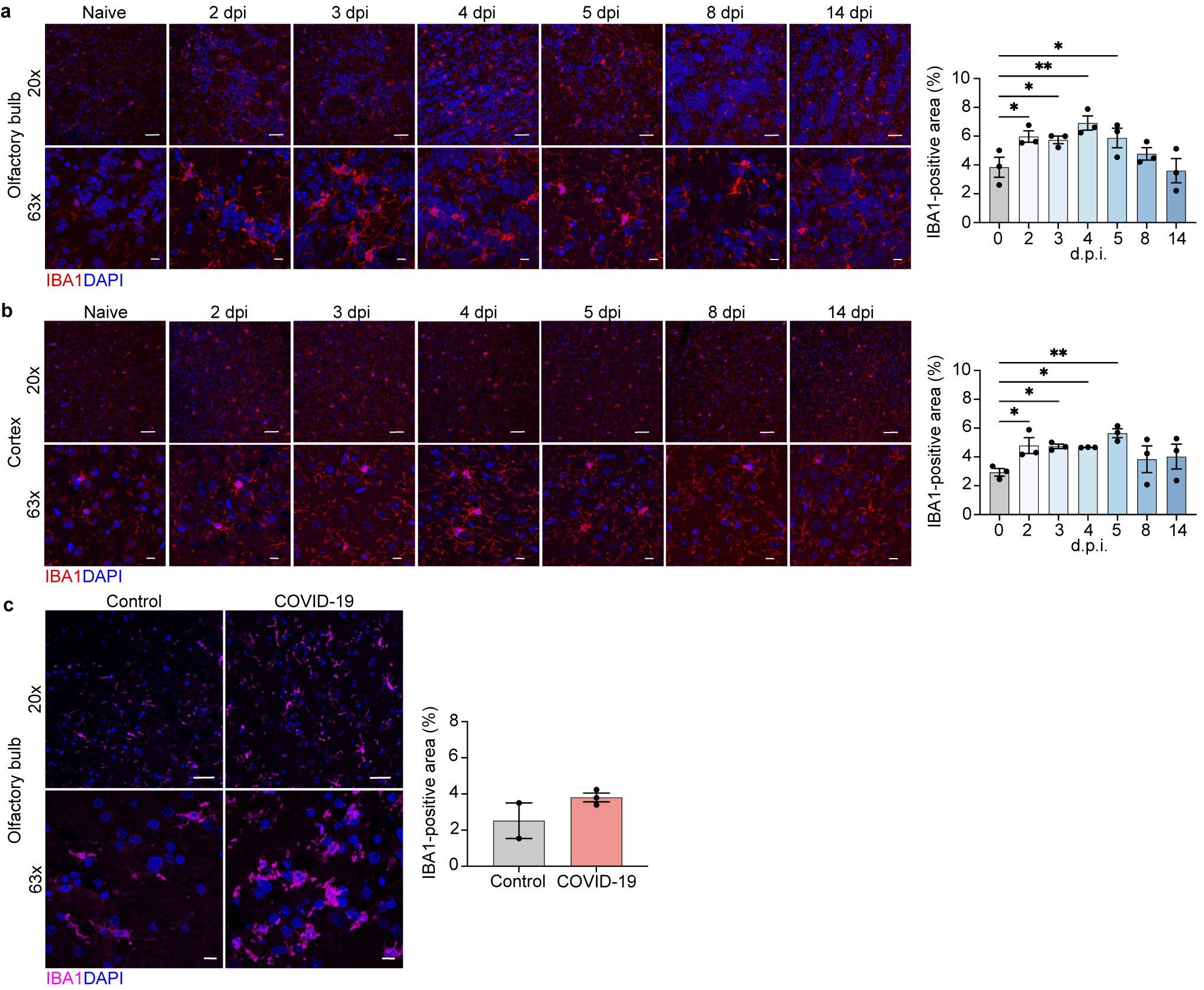

Supplement: Supplement 8 [file c4d709c41ecfebef775c9a68.tif]

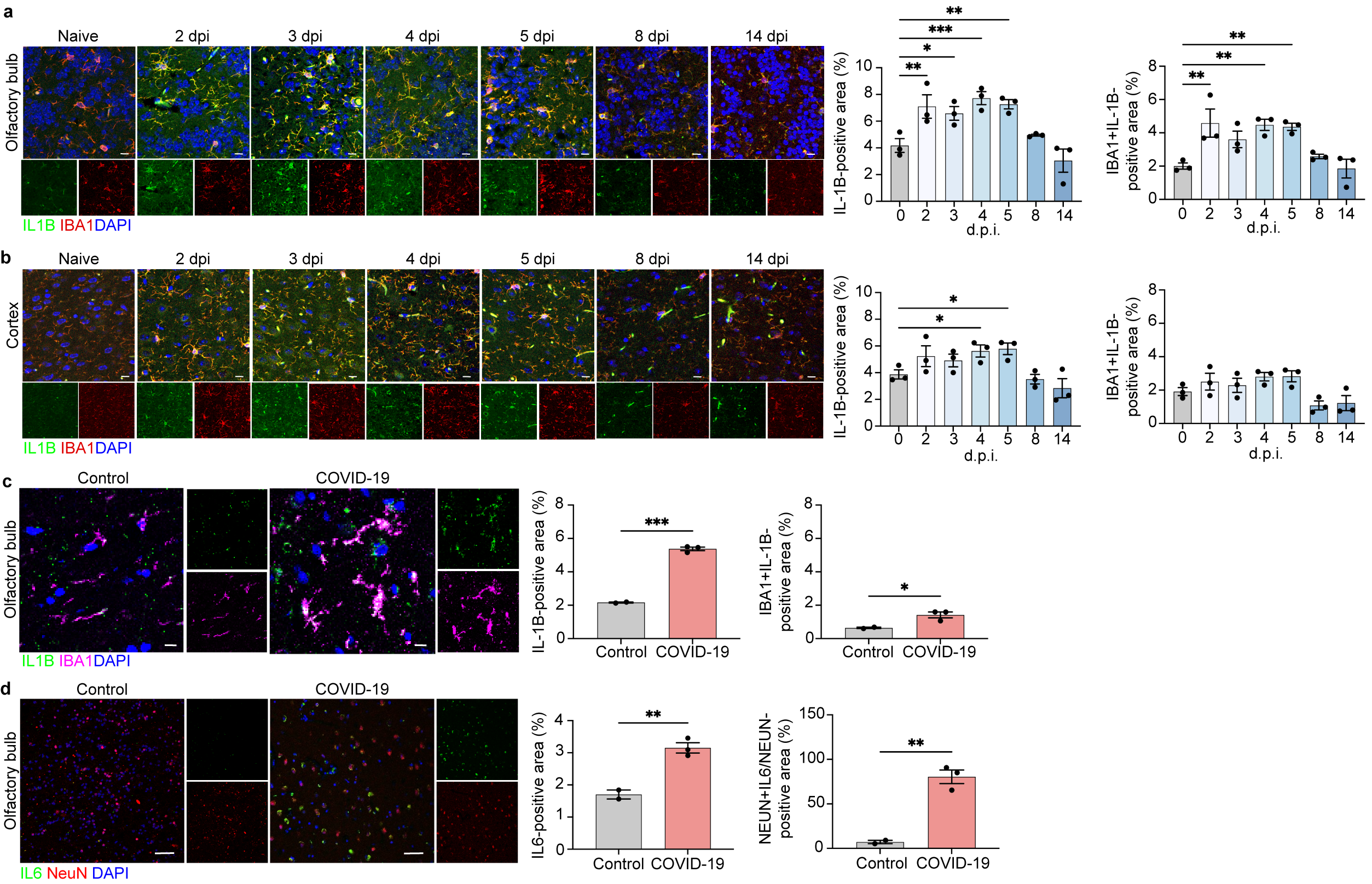

Supplement: Supplement 9 [file 0c5f62877a6d24834427f746.tif]

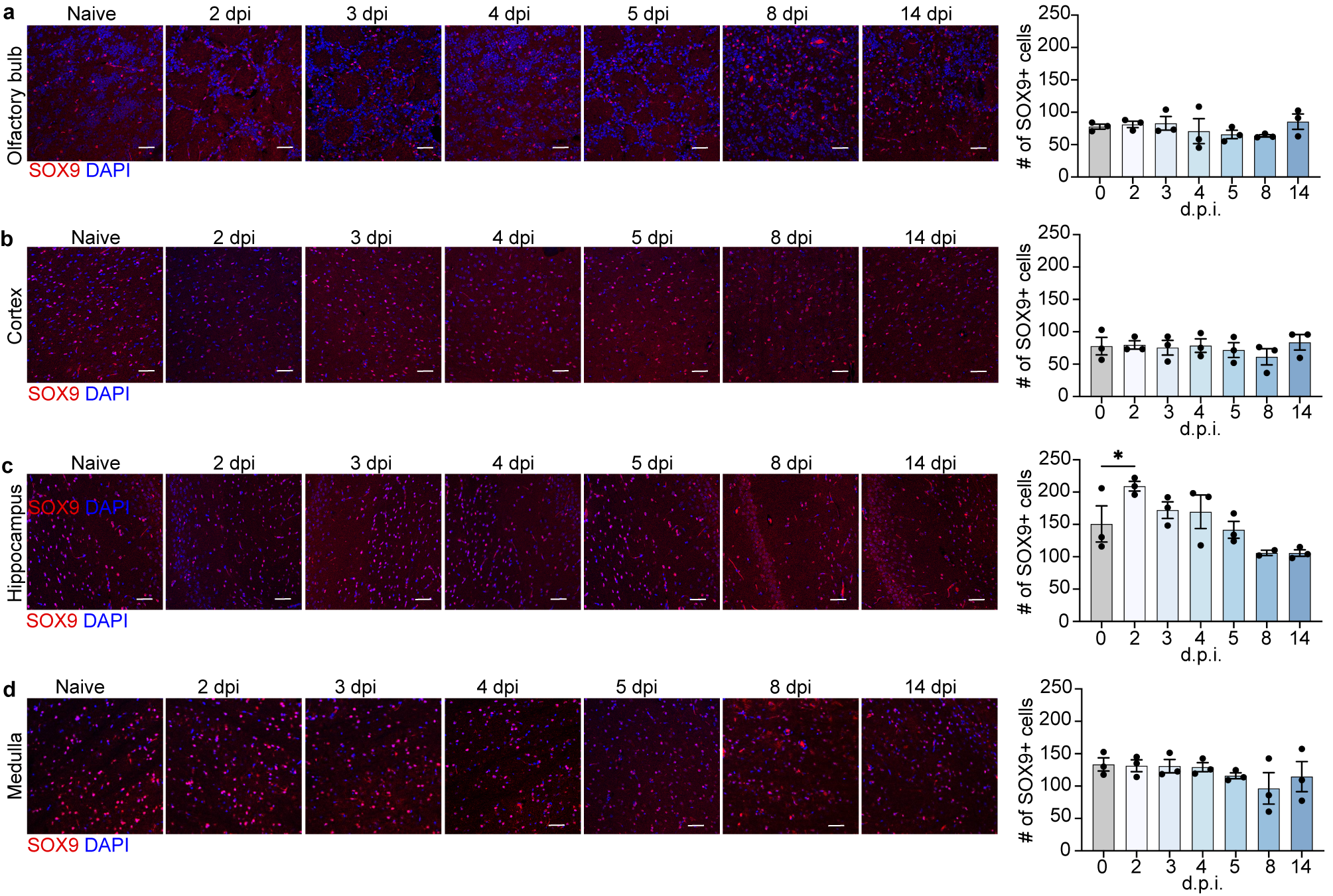

Supplement: Supplement 10 [file 145a8023cb1c6e9a01f9ebf9.tif]

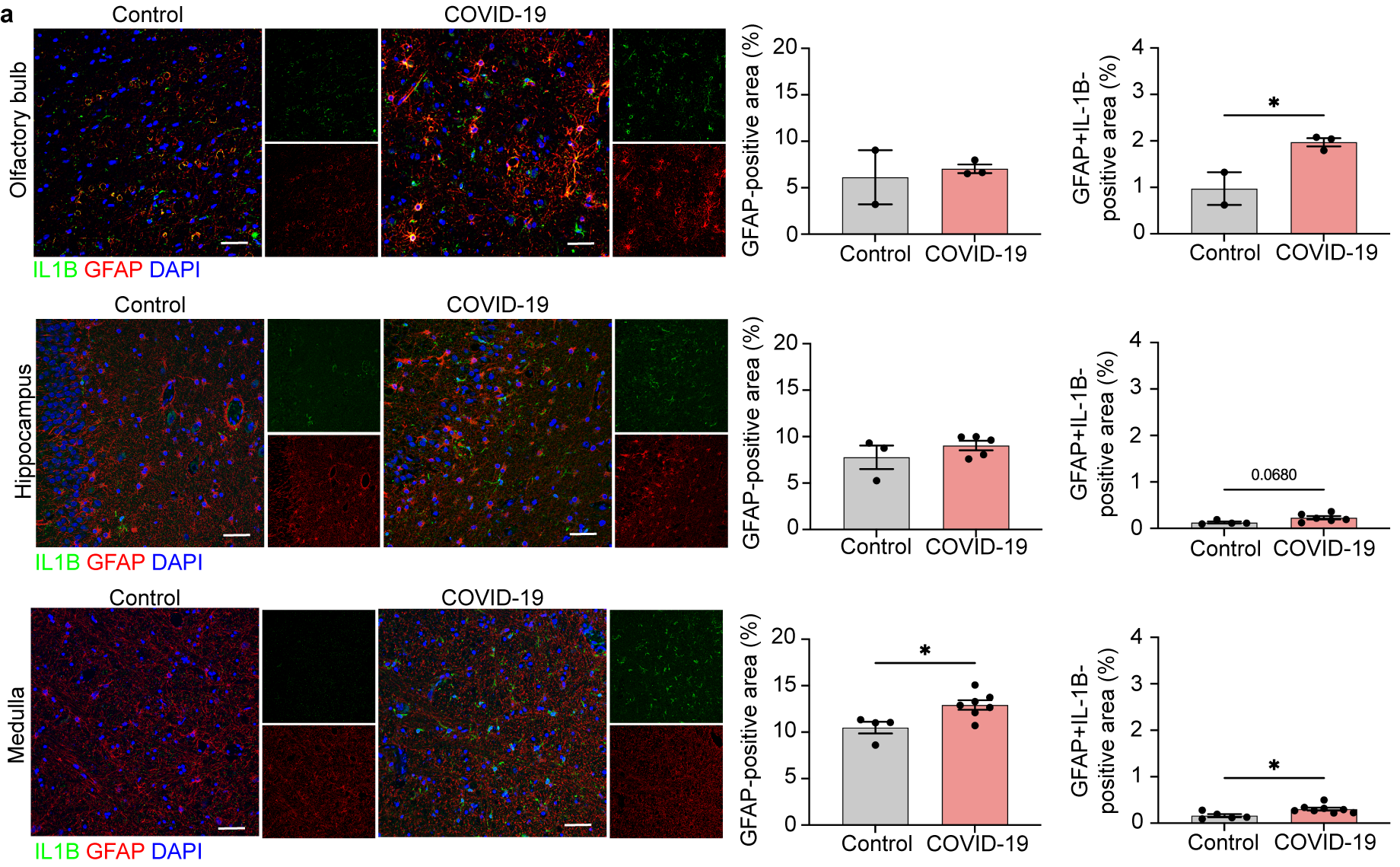

Supplement: Supplement 11 [file f0121f1840d15ee49240b119.tif]

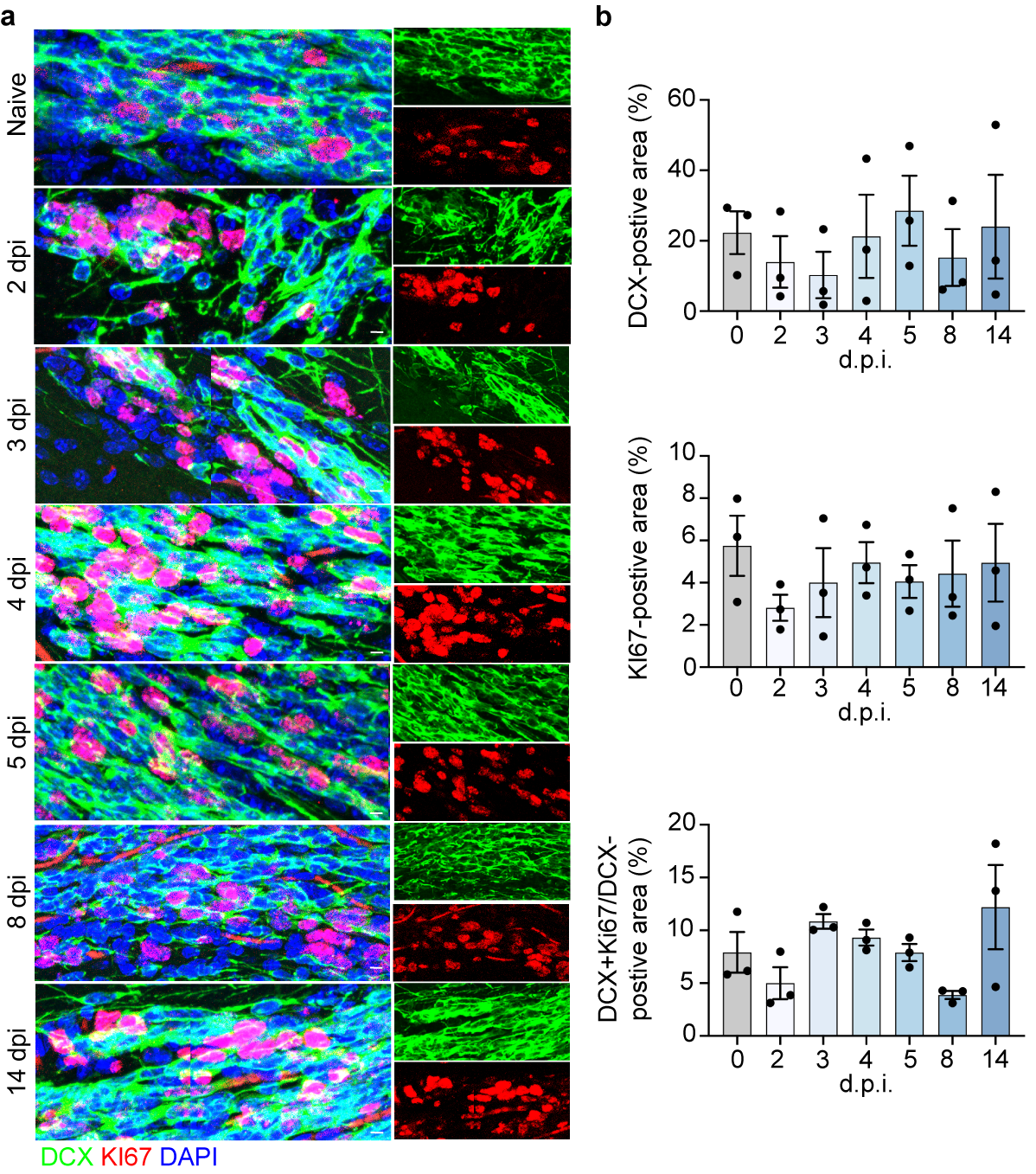

Supplement: Supplement 12 [file 9c41a238652b7f01343c8c2d.tif]

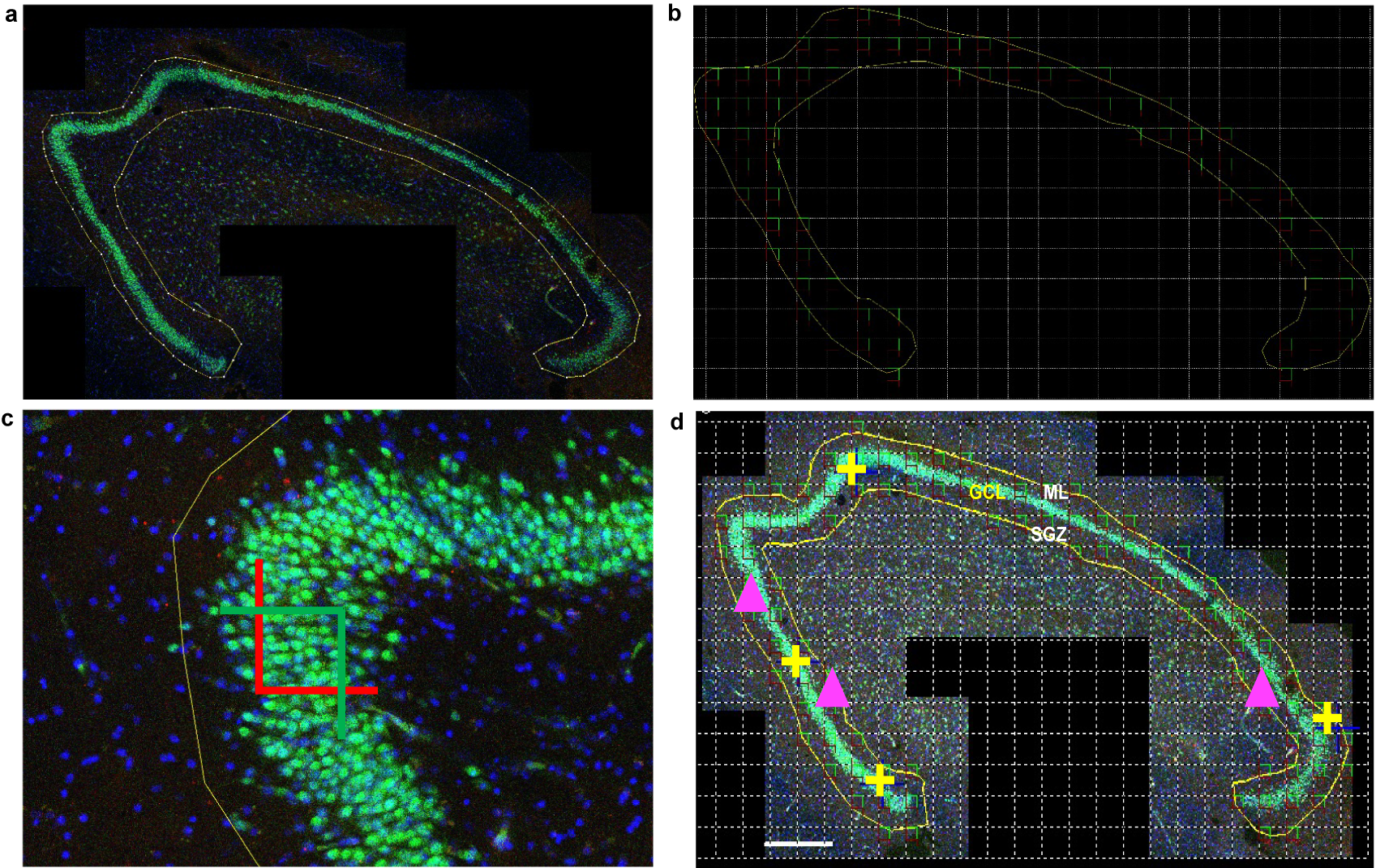

Supplement: Supplement 13 [file f304c7795f0daa634d95ecd8.tif]

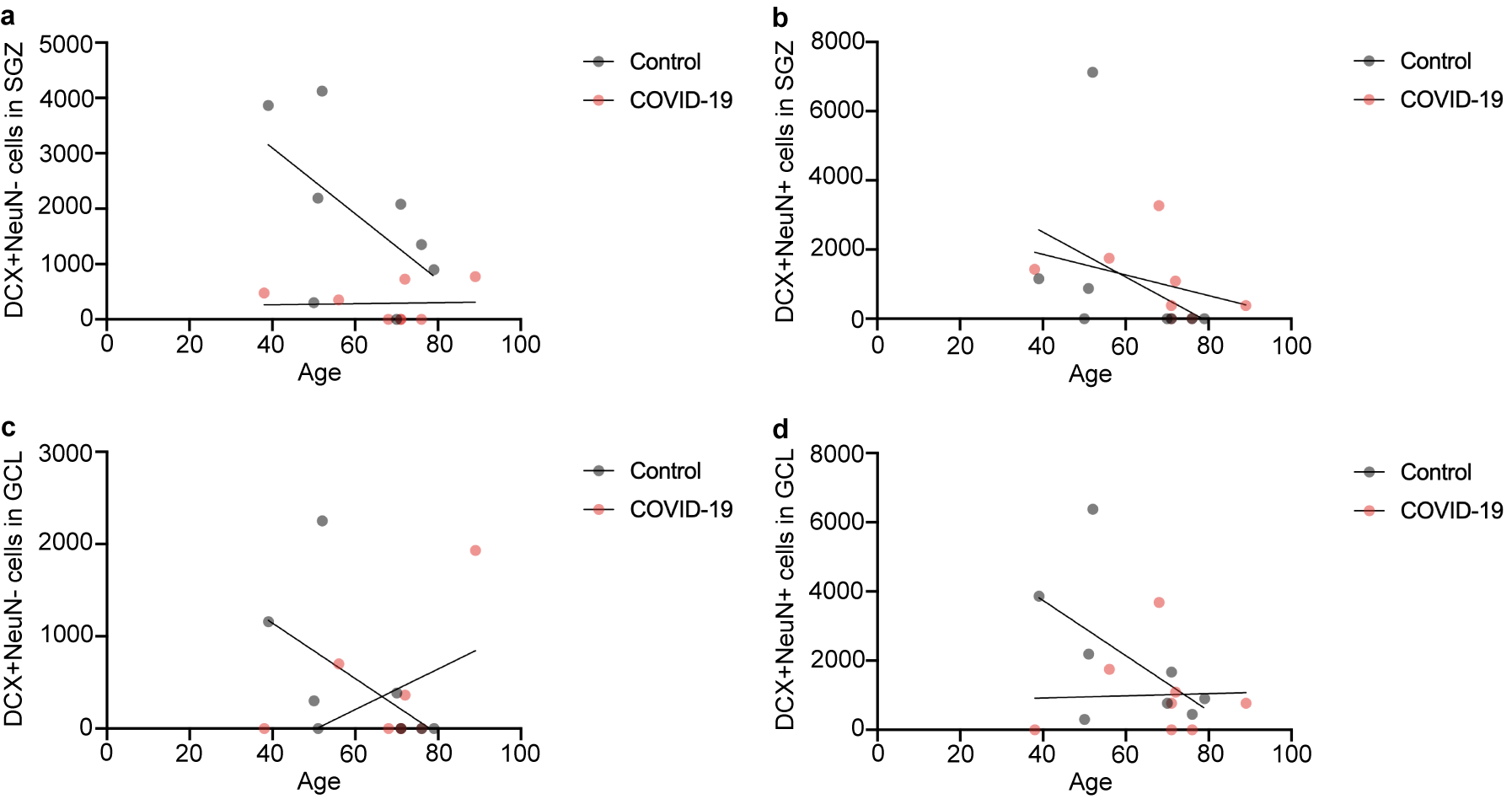

Supplement: Supplement 14 [file 9a9c6c65213f98c0d9873733.tif]
